# The impact of simulation-based trabeculectomy training on resident core surgical skill competency

**DOI:** 10.1097/IJG.0000000000002114

**Published:** 2022-08-22

**Authors:** Roxanne Annoh, John Buchan, Stephen Gichuhi, Heiko Philippin, Simon Arunga, Agrippa Mukome, Fisseha Admassu, Karinya Lewis, William Makupa, Juliet Otiti-Sengeri, Min Kim, David MacLeod, Matthew J. Burton, William H. Dean

**Affiliations:** 1 International Centre for Eye Health, Department of Clinical Research, London School of Hygiene and Tropical Medicine, London, UK; 2 Department of Ophthalmology, University of Nairobi, Kenya; 3 Eye Centre, Medical Centre, Faculty of Medicine, University of Freiburg, Germany; 4 Mbarara University & Referral Hospital Eye Centre (MURHEC), Mbarara University of Science and Technology, Mbarara, Uganda; 5 Department of Ophthalmology, Parirenyatwa Hospitals, University of Zimbabwe, Harare; 6 University of Gondar, Ethiopia; 7 Ophthalmology Department, Salisbury Hospitals NHS Foundation Trust, UK; 8 Eye Foundation Hospitals, Lagos, Nigeria; 9 Kilimanjaro Christian Medical Centre, Moshi, Tanzania; 10 Department of Ophthalmology, School of Medicine, Makerere University, Kampala, Uganda; 11 Tropical Epidemiology Group, Faculty of Infectious Disease Epidemiology, London School of Hygiene and Tropical Medicine, London; UK; 12 National Institute for Health Research Biomedical Research Centre for Ophthalmology at Moorfields Eye Hospital NHS Foundation Trust and UCL Institute of Ophthalmology, United Kingdom; 13 Division of Ophthalmology, University of Cape Town, South Africa

**Keywords:** Ophthalmology, Training, Africa, Simulation, Education, Glaucoma

## Abstract

**Purpose:**

To measure the impact of trabeculectomy surgical simulation training on core surgical skill competency in resident ophthalmologists.

**Methods:**

This is a post-hoc analysis of the GLAucoma Simulated Surgery (GLASS) trial, which is a multi-center, multi-national randomized controlled trial. Resident ophthalmologists from six training centers in sub-Saharan Africa (in Kenya, Uganda, Tanzania, Zimbabwe and South Africa) were recruited according to the inclusion criteria of having performed zero surgical trabeculectomies and assisted in less than five. Participants were randomly assigned to intervention and control arms using allocation concealment. The intervention was a one-week intensive trabeculectomy surgical simulation course. Outcome measures were mean surgical competency scores in eight key trabeculectomy surgical skills (scleral incision, scleral flap, releasable suturing, conjunctival suturing, sclerostomy, tissue handling, fluidity and speed), using a validated scoring tool.

**Results:**

Forty-nine residents were included in the intention-to-treat analysis. Baseline characteristics were balanced between arms. Median baseline surgical competency scores were 2.88/16 (IQR 1.75-4.17) and 3.25/16 (IQR 1.83-4.75) in the intervention and control arms respectively. At primary intervention, median scores increased to 11.67/16 (IQR 9.58-12.63) and this effect was maintained at three months and one year (p= 0.0001). Maximum competency scores at primary intervention were achieved in the core trabeculectomy skills of releasable suturing (n=17, 74%), scleral flap formation (n=16, 70%) and scleral incision (n=15, 65%) compared to scores at baseline.

**Conclusion:**

This study demonstrates the positive impact of intensive simulation-based surgical education on core trabeculectomy skill development. The rapid and sustained effect of resident skill acquisition pose strong arguments for its formal integration into ophthalmic surgical education.

## Introduction

Glaucoma is the leading cause of irreversible blindness, affecting approximately 76 million people worldwide in 2020.^1^ Recent estimates suggest that over 100 million people will be diagnosed with the condition by 2040, largely due to an increasing and ageing global population.^1^ Primary openangle glaucoma (POAG), in particular, develops earlier in those of African ancestry, with a more aggressive and rapid progression to advanced disease compared to other ethnic groups.^2^ Currently, Africa has the highest global prevalence of glaucoma and POAG, estimated at 4.8 % (95% CI 2.6- 8.0) and 4.2% (CI 2.1-7.4) respectively in those aged 40 to 80 years.^1^ Importantly, glaucoma is responsible for 4.4% (CI 4.1- 5.0) of all blindness in Africa, which is proportionately much higher than other regions in the world.^3^ Urgent public health measures are therefore required to control and reduce the disease burden in the region.

The most effective way to slow the rate of disease progression in glaucoma is by targeted lowering of intraocular pressure (IOP). This is typically done using a step-wise approach, depending on the subtype of disease, the extent of optic nerve damage, and the degree of visual field dysfunction. Yet, in sub-Saharan Africa (SSA), management of glaucoma is challenging for several reasons; late diagnosis, poor adherence to treatment and limited access to healthcare services and treatment. ^4–6^ Furthermore, a profound lack of patient awareness about the condition means that those affected often present with advanced, irreversible sight loss. In POAG, the initial step in treatment is medical, using long-term topical drop therapy, but in SSA, this is confounded by barriers in affordability, adherence and side effects. ^5,7^ Laser therapy, such as selective laser trabeculoplasty, is effective in lowering IOP and offers an alternative and safe initial treatment for African individuals with mild to moderate glaucoma. ^8,9^ However, the IOP lowering effect from laser treatment is only temporary, with many requiring repetitive treatment or initiation of medical or surgical therapy later in life. Furthermore, its efficacy in advanced disease remains unclear. For these reasons, surgical management of glaucoma, in the form of trabeculectomy, is often recommended as the first line choice in SSA.^10^

Trabeculectomy remains the gold standard surgical procedure and is the most effective technique for long-term IOP management. ^7,11^ However, the provision of trabeculectomy depends on the availability of ophthalmic surgeons with surgical proficiency. At present, there is a global shortage of ophthalmologists, with a disproportionate shortage of ophthalmologists in SSA (an average ratio of 2.5 per million population, against a global average of 31.7) that are mostly confined to urban areas.^12,13^ Due to the magnitude of disease burden and high general patient workload, ophthalmologists are often denied the opportunity for sub-specialist training in glaucoma, resulting in a paucity of glaucoma surgical skills.^12^ These challenges, coupled with a low uptake of surgical treatment, a fear of surgical complications and challenges in post-operative care, make many ophthalmologists reluctant to offer trabeculectomy as a first line treatment option to patients. ^12,14^

A practical solution is to enhance the existing surgical skillset of current and prospective ophthalmologists in SSA. There is widespread variability in the number of trabeculectomies performed during residency, with a mean of 4 (median of 1) in a recent survey of resident ophthalmologists in the Eastern, Central and Southern African (ECSA) region.^15^ Qualitative analysis found that residents in the region expressed a need for improvement in conventional ophthalmic surgical training, with better supervision and more use of simulation-based surgical education (SBSE). ^15^ Conventional ophthalmic surgical teaching in SSA typically uses theoretical-based learning, observation, low use of SBSE (mostly using low to moderate-fidelity simulation models), followed by live surgical teaching for advanced skill development.^16^ Importantly, the use of SBSE varies across the different training institutions in the region and is not uniformly integrated into ophthalmic surgical training. For those using SBSE in their ophthalmic surgical training, many report inadequacy of training facilities and tools, as well as a lack of trainer supervision.^16^ Yet, compared to the traditional Halstedian apprenticeship model of “see one, do one, teach one”, SBSE offers a safer alternative for junior surgeons to refine their skills in the absence of patient harm, by using artificial training models. It is associated with less error rate, improvement in skill acquisition and fewer intraoperative complications.^17-20^ Yet, whilst there is extensive research in simulation techniques for cataract surgery, data on SBSE in glaucoma surgery is limited. At the time of writing, there is no known integrated, comprehensive SBSE course on surgical trabeculectomy in SSA. The GLAucoma Simulated Surgery (GLASS) trial is the first known randomised controlled trial (RCT) assessing the efficacy of intense SBSE in glaucoma surgery on overall surgical competence, confidence, and live trabeculectomy surgery output in SSA-based resident ophthalmologists. ^21^ Here we present a post-hoc analysis of the GLASS trial data that evaluates the impact of SBSE on core trabeculectomy surgical skill competency in resident ophthalmologists.

## Materials & Methods

### Study Design

This is a post-hoc analysis from the GLASS trial, which is a randomised controlled, parallel-group efficacy trial conducted between October 2017 and July 2019. Trial participants were randomized to two arms, with intended 1:1 allocation ratio. The trial design and primary results have been fully presented elsewhere. ^21,22^ Ethical approval was obtained from the London School of Hygiene and Tropical Medicine and the collaborating research institutions.^21,22^ The trial was registered (PACTR201803002159198).

### Setting & Participants

Resident ophthalmologists from six training centers in Kenya, Uganda, Tanzania, Zimbabwe and South Africa, were recruited according to the inclusion criteria of having performed zero surgical trabeculectomies and assisted in less than five. Participants were in their second, third or fourth year of postgraduate ophthalmology training.

### Intervention

The trial intervention was a one-week, intense trabeculectomy SBSE course. The course consisted of theoretical and practical-based teaching on glaucoma and trabeculectomy surgery. The surgical procedure was deconstructed and instruction in surgical steps was provided using a modified Peyton’s four-stage approach.^21,23^ Individual steps of the procedure were practiced using low cost, moderate-fidelity simulation materials including foam materials for suturing practice and apple peels for scleral flap construction.^24^ A full trabeculectomy procedure was performed on high-fidelity synthetic ‘Advanced TrabEye’ simulation surgery eyes (PS-023, Phillips Studio, Bristol, UK) and using Zeiss Stemi 305 microscopes (Carl Zeiss Microscopy, Jena, Germany) for the competency assessments. Each resident’s trabeculectomy procedure on the high-fidelity synthetic ‘Advanced TrabEye’ was recorded using the Zeiss Labscope App (V.2.8.1) on iPads. Participants allocated to the control arm received the exact same intervention shortly after the one year follow-up assessment.

### Outcomes

**Figure 1:**
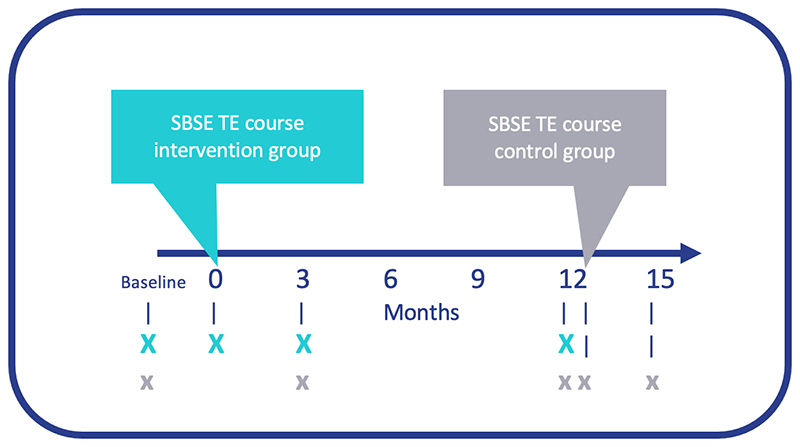
Timeline of interventions (simulation-based surgical education trabeculectomy (SBSE TE) course) and assessments of the primary intervention (X) and intervention in the control (x) groups using the Sim-OSSCAR tool. Sim:OSSCAR =Ophthalmic Simulated Surgical Competency Assessment Rubric^25^

Participants were assessed on their competency in completing a full trabeculectomy procedure using the ophthalmic simulated surgical competency assessment rubric (Sim-OSSCAR) grading tool. ^25^ Timelines of assessment were at baseline, primary intervention (time of intervention in the intervention arm), three months, one year, time of intervention in the control arm, and fifteen months (equivalent to three months after intervention received in the control arm, Figure 1).

**Figure 2:**
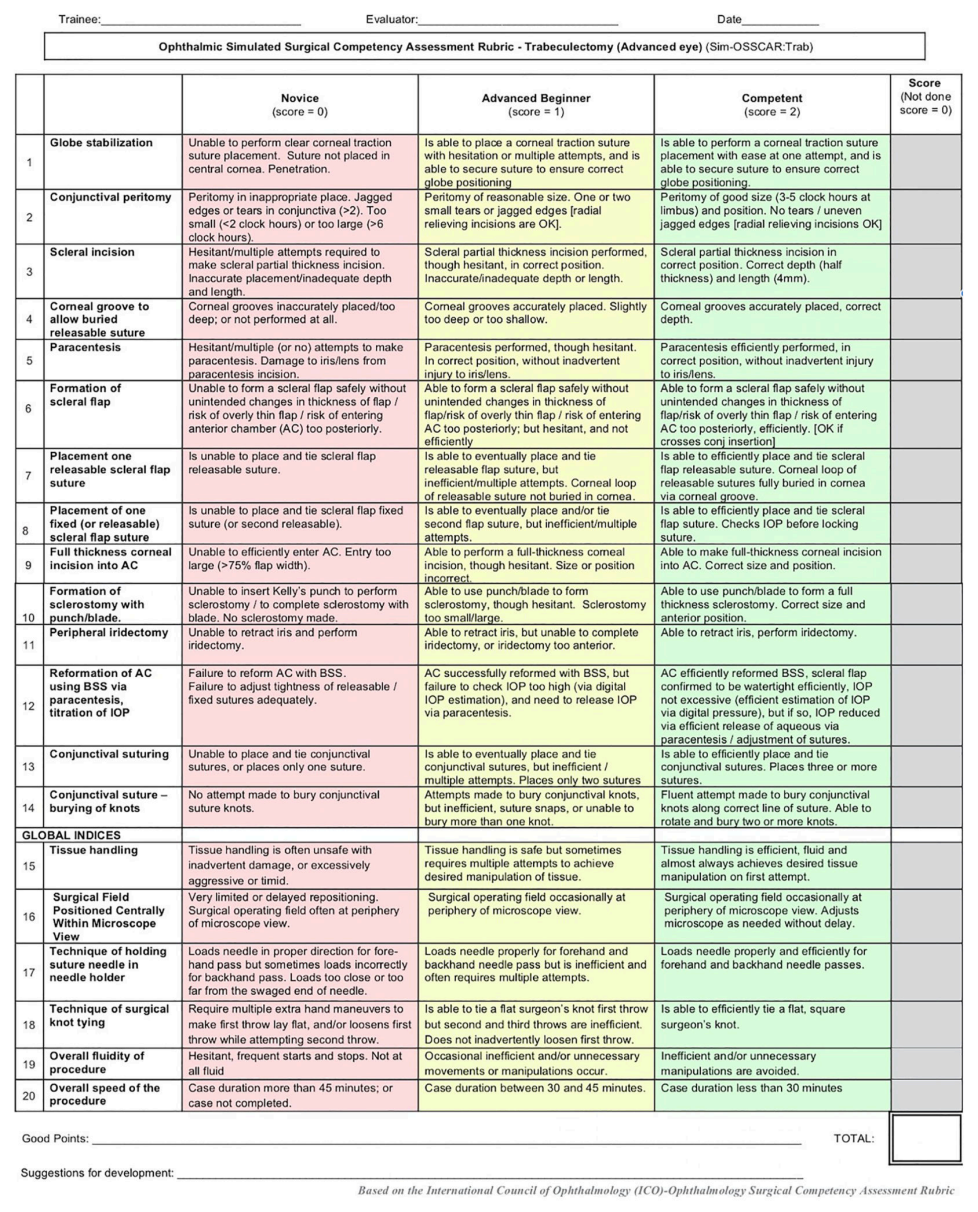
Sim:OSSCAR tool for simulated trabeculectomy. Performance of each individual core surgical skill is ranked from 0 (novice), 1 (advanced beginner) and 2 (competent). Sim:OSSCAR =Ophthalmic Simulated Surgical Competency Assessment Rubric

**Figure 3:**
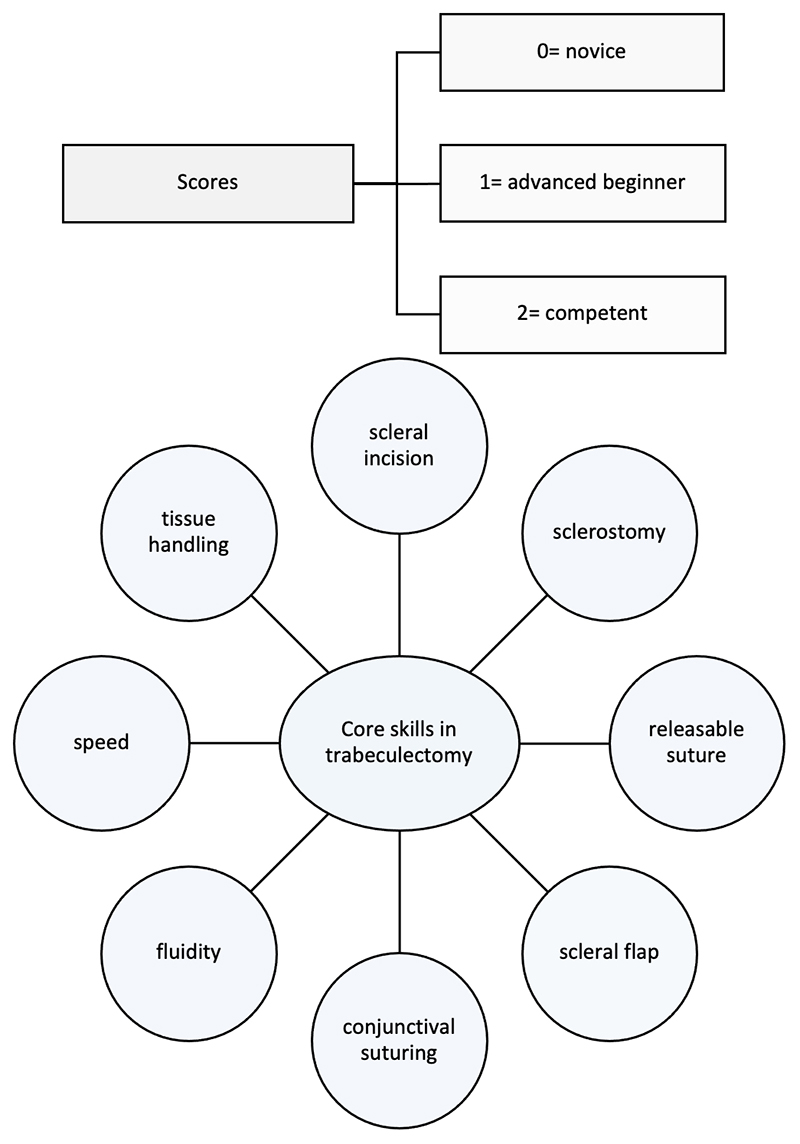
The GLASS trial core skills in trabeculectomy and assessment scores. GLASS= GLAucoma Simulated Surgery, Sim:OSSCAR= Ophthalmic Simulated Surgical Competency Assessment Rubric

Anonymized video recordings of the procedures were assessed by two independent, masked graders who were experts in glaucoma surgery and had undergone familiarization training using the Sim-OSSCAR tool (Figure 2). Video recordings of procedures were allocated a random seven-digit number, being the only identifiable information available for grading. Each grader was therefore fully masked to the participant’s identity, allocation arm, training institution and timing of surgical assessment. The primary outcome measure was the combined mean score of three masked assessments of simulation surgical performance over the study period in eight selected core skills from the Sim-OSSCAR tool (Figure 3). Each grader evaluated a minimum of two and maximum of three anonymized videos, and allocated a maximum score of 2 to each selected core surgical skill. The maximum overall score for the combined surgical skills per anonymised video was 16. Secondary outcome measures included individual core surgical skill competency scores most improved after intervention and the trends in individual core surgical skill competency scores over the 15 month study period.

### Statistical analysis

The GLASS trial protocol and primary analysis included the sampling strategy, sample size and power calculations. ^21,22^ Intention-to-treat (ITT) analysis was used for all outcome measures. Results were presented as mean ± standard deviation (SD) for parametric data, and median and interquartile range (IQR) for non-parametric data. Wilcoxon signed rank test was used for differences in combined core skill competency scores at each assessment timeline and for differences in scores between trial arms. Residents achieving maximum scores in competency in individual core surgical skill were presented as numbers and percentages, with Fisher’s exact test used to measure statistical significance for differences in proportions between trial arms and McNemar’s test for differences at each assessment timeline. All statistical analyses were conducted using STATA for Windows version 16.0 (StataCorps, Texas, USA), with an alpha level of p<0.05 deemed as statistically significant.

## Results

Fifty-three participants were assessed for eligibility for the GLASS trial during the study period. Two participants were excluded pre-randomisation due to prior surgical experience. Fifty-one participants were recruited and randomised, with 25 allocated to the intervention arm with two dropouts, and 26 to the control arm. Forty-nine participants were included in the GLASS trial ITT sub-analysis^21^, in whom baseline characteristics of age, sex and time in residency were balanced.

### Overall surgical competency in simulated trabeculectomy

The median combined surgical competency scores at baseline were 2.88/16 points (IQR 1.75-4.17) and 3.25 (IQR 1.83-4.75) in the intervention and control arms respectively (Table 1). At primary intervention, median scores increased to 11.67 (IQR 9.58-12.63; p=0.00001). This increase in core surgical competency scores was maintained at three months (median 11.67, IQR 10.33-13.17; p=0.00001) and at one year (median 11.50, IQR 9.67-12.67; p=0.0001) in the intervention arm. On receiving the intervention after one year of conventional training, median scores in the control arm increased to 11.33 (IQR 10.67-12.50; p=0.00001). The increase was maintained at fifteen months (median 11.00, IQR 8.17-14.00; p=0.0156). When comparing the trial arms, the difference between combined surgical competency scores at three months and at one year showed a large effect of the training intervention (p=0.00001, Table 2).

### Trends in surgical skill competency over time

**Figure 4:**
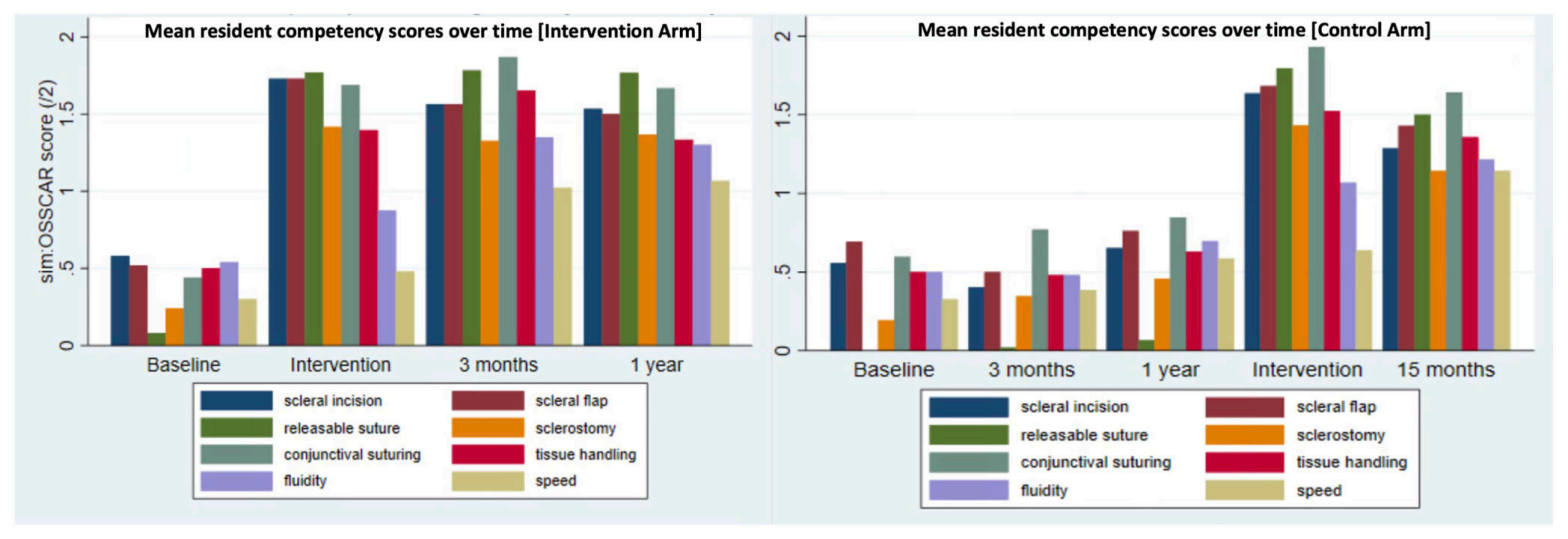
Mean resident competency scores by core surgical skill between arms, over time. Primary intervention denotes simulation training in the intervention arm, given shortly after baseline assessment. Intervention in the control arm occurred shortly after one year. Sim:OSSCAR= Ophthalmic Simulated Surgical Competency Assessment Rubric

Figure 4 illustrates the mean scores of individual core surgical skill between arms. Trial participants in both arms achieved higher mean scores in core surgical skill competency on receiving the intervention. In the intervention arm, the highest score achieved was in releasable suturing at primary intervention (mean 1.77± SD 0.42). At three months, the highest score was in conjunctival suturing (mean 1.87± SD 0.22); at one year, the highest score was releasable suturing (mean 1.71± SD 0.30). Conversely, mean scores in the control arm at three months and one year were similar to those at baseline. Following intervention in the control arm at one year, the highest score was seen in conjunctival suturing (mean 1.93 ±SD 0.23) and remained so at 15 months (mean 1.64 ±SD 0.38). Lowest scores were achieved in speed at primary intervention (mean 0.48 ±SD 0.71) and remained so at three months (mean 1.02 ± SD 0.71) and at one year (mean 1.03 ±SD 0.72) in the intervention arm. The lowest scores were also in speed in the control arm at the time of intervention and at 15 months (mean 0.64 ± SD 0.64 and mean 1.14 ± SD 0.69 respectively).

### Maximum scores in surgical skill competency

**Figure 5:**
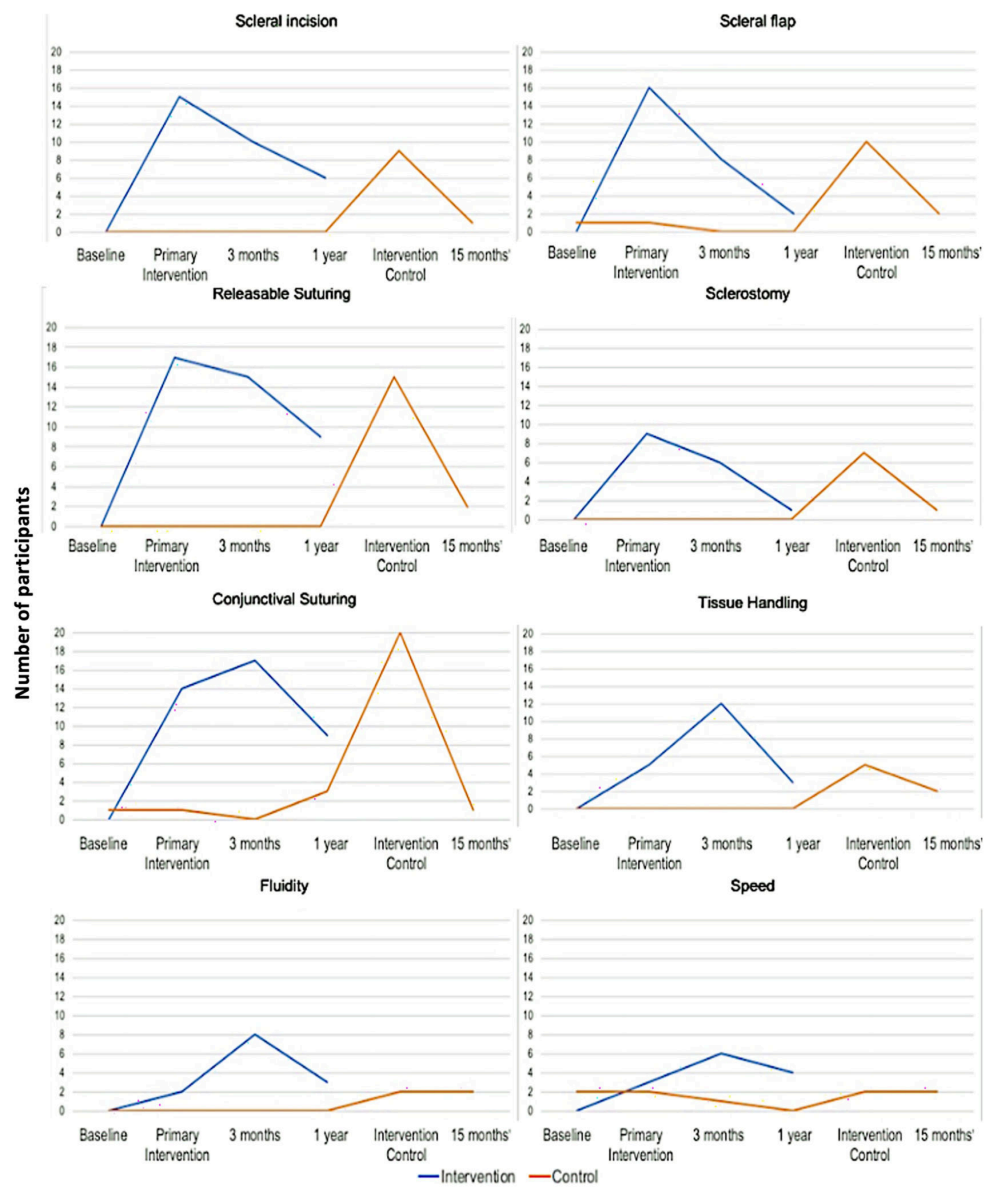
Number of participants achieving maximum scores in competency in surgical skills over time

Few participants achieved maximum scores in surgical skill competency prior to receiving the intervention (Figure 5). At primary intervention, releasable suturing was the most competent skill achieved, with 17/23 (74%) participants in the intervention arm achieving maximum scores. This was followed by scleral flap (n=16, 70%) and scleral incision (n=15, 65%). However, the number of participants with maximum scores declined at three months and again at one year. The exception was in fluidity and speed, where participants achieving maximum scores in these skills were significantly higher at three months than at the time of primary intervention (χ^2^ = 6.53, p=0.0106 and χ^2^= 8.33, p=0.0039 respectively, McNemar’s test). The number of participants in the control arm achieving maximum scores rose from one (4%) at three months to three (13%) at one year. Following the intervention, 20 participants (83 %) achieved maximum scores in conjunctival suturing, followed by releasable suturing (n=15, 63%) and scleral flap (n=10, 42%). When comparing the two arms, only maximum scores in conjunctival suturing were significantly different (p=0.018, Fisher’s exact), with the control arm achieving more maximum scores after intervention than the intervention arm had at that same point.

## Discussion

### Overall efficacy of glaucoma surgical simulation

The GLASS trial is the first known international multi-center RCT to demonstrate a positive effect of glaucoma surgical simulation training on surgical competency of ophthalmology residents.^21^ Participants in both the control and intervention arms showed significant improvement in competency after receiving high-fidelity, intense SBSE and this effect was maintained months after the intervention. There was a significant difference in competency between the trial arms, illustrating the disparity in skill uptake between those receiving conventional ophthalmic surgical teaching and simulation-based training. Few studies are available for direct comparison. A study evaluating the efficacy of virtual-reality (VR) SBSE on resident and medical student competency in simulated pars plana vitrectomy found that those naïve to simulation had longer operating times and more incidences of retinal detachments compared to those with simulation training.^26^ However, these findings were not statistically significant, owing to a low sample size of 14. Similarly, Solverson et al. reported marked improvement in the error rate of novice surgeons using the Eyesi VR simulator, yet the study lacked a simulation naïve comparison group or a validated means of skill assessment.^27^ As the GLASS trial used a validated scoring rubric and adopted an RCT study design, our findings strongly indicate that SBSE can have an immediate and sustained improvement in glaucoma surgical skills.

### Efficacy of glaucoma surgical simulation on core surgical skills

In the absence of training, residents scored poorly in core skills required for modern trabeculectomy surgery such as releasable suturing. Conversely, they scored highest in scleral incision and flap formation, possibly from previous surgical experience in small incision cataract surgery.^15^ With conventional training alone, mean scores remained at novice level, with little progression to competent level. Yet, a significant and sustained improvement in competency was observed in both arms shortly after receiving simulation training. Importantly, skills traditionally used in trabeculectomy surgery, such as releasable suturing, sclerostomy and conjunctival suturing, saw the biggest improvement overall which supports the hypothesis that targeted simulation training can refine sub-specialist surgical skills. Of note, there was little change in general skills such as fluidity and speed possibly due to insufficient repetitive skill practice by residents over the course of the study. Continuous simulation practice may therefore help reduce overall trabeculectomy surgery time.

Although glaucoma SBSE led more residents to progress to competent level, the subsequent decline in competent scores in later months suggests that residents may become deskilled in acquired trabeculectomy surgical skills over time. This may be due to insufficient exposure to live trabeculectomy surgery practice in conventional training or inadequate uptake of simulation practice between follow-up assessments. Arguably, SBSE should be used to complement traditional surgical teaching rather than replace it ^28^, as transfer of surgical skills to the operating room can vary widely depending on the type and amount of simulation training received.^19,27^ Moreover, the true association between simulated training and clinical practice remains uncertain. When examining transfer of skills to live surgery, most studies have adopted a retrospective study design to investigate the effects of simulation training based on patient outcomes. For example, one US-based retrospective case series found significantly lower phacoemulsification complication rates in residents with VR simulation training compared to simulation-naïve residents (2.4% vs 5.1% respectively, p=0.037).^29^ However, Belyea et al’s retrospective case review reported no significant difference in phacoemulsification complication rates between third year residents with and without VR simulation training.^19^ Prospective assessment and comparison of surgical skill competency in both simulated and live surgeries may be useful to determine the true effect of simulation training.

#### Limitations

This study has several limitations. Firstly, this is a retrospective post-hoc analysis of data from the GLASS trial. Therefore, the GLASS trial was not originally powered to address the hypothesis that specific skills benefit more from intense simulated-based training in trabeculectomy. As a result, the true efficacy of the intervention reported in our study may be exaggerated due to sub-analyses of the original data producing falsely positive and/or negative associations between the variables. Secondly, whilst this study clearly shows superiority of glaucoma surgical simulation training over conventional training, the results only apply to simulated surgical skill competency using high-fidelity artificial eyes. In SSA, a comparison and evaluation of ophthalmic SBSE between high and low-fidelity models would be beneficial for reflecting clinical practice in low- and middle-income settings. Due to low trabeculectomy case numbers in the respective training environments, it was also not possible to evaluate and compare live surgical skills with those in the simulated environment. Furthermore, the low response rate in the control group at fifteen months (n=7, 26.9%) makes the findings susceptible to selection bias, distorting the true measure of effect of the intervention. This low response rate was due to trial participants completing their Master of Medicine (MMed) in Ophthalmology degree and no longer being able to participate in the study. Comparison of post intervention scores between the trial arms should also therefore be interpreted with caution. Finally, further detailed analysis of participants failing to achieve “advanced beginner” or “competency” Sim:OSSCAR scores following the intervention would have been beneficial to evaluate how best to refine the intervention to improve their skillset.

## Conclusion

This study is the first to show a positive, immediate and sustained impact of SBSE on key and core surgical skills in trabeculectomy. Trabeculectomy remains the most effective surgical treatment for glaucoma management in SSA but performing the procedure requires advanced microsurgical skills. Evaluating the performance of each surgical step is essential for providing targeted, constructive feedback to residents. Time taken to complete a task is a commonly used outcome measure for SBSE studies,^30^ however our findings suggest that the outcome measure of speed is not the best indicator of impact. Formal integration of glaucoma surgical simulation into residency programme structures may result in better standards of surgical training and most importantly, improve the delivery of safe and effective glaucoma surgical treatment. Recent observations suggest that adopting surgical simulation training is widely accepted as a safer alternative to conventional surgical teaching. ^16^ Finally, there remains very limited data on surgical trabeculectomy rates and post-operative outcomes in SSA. We therefore suggest a follow-up comprehensive, comparative analysis of trabeculectomy outcomes in centers incorporating SBSE, to evaluate the real world effectiveness of the intervention on patient glaucoma care.

## Figures and Tables

**Table 1:** Combined surgical competency scores over the GLASS trial study period, in each trial arm.

Intervention arm	Timeline	n	median	IQR	p value
	**Baseline**	25	2.88	1.75-4.17	
	**Primary intervention**	23	11.67	9.58-12.63	0.00001*
	**3 months**	23	11.67	10.33-13.17	0.00001*
	**1 year**	19	11.50	9.67-12.67	0.0001*
Control arm	Baseline	26	3.25	1.83-4.75	
	**3 months**	26	3.67	2.67-5.00	0.1443
	1 year	24	4.17	3.33-5.83	0.0319
	At intervention	24	11.33	10.67-12.50	0.00001*
	15 months	7	11.00	8.17-14.00	0.0156*

* p<0.05 using Wilcoxon signed rank test in reference to baseline scores. n= number, IQR= interquartile range, GLASS=GLAucoma Simulated Surgery

**Table 2:** Combined surgical competency scores over the GLASS trial study period, between trial arms.

Timeline	Control			Intervention			p
	n	median	IQR	n	median	IQR	
**Baseline**	26	3.25	1.83-4.75	25	3.00	1.83-4.33	0.8923
**3 months**	26	3.67	2.67-5.00	23	11.67	10.33- 13.17	0.00001*
**1 year**	24	4.17	3.50-5.83	19	11.67	9.83-12.67	0.00001*
**At Intervention**	24	11.33	10.67-12.50	23	11.67	9.58-12.63	0.9176

* p <0.05 using Wilcoxon rank sum test for comparison between arms. 15 months excluded from analysis. n= number, IQR= interquartile range, GLASS=GLAucoma Simulated Surgery

## References

[1] Tham YC, Li X, Wong TY, Quigley HA, Aung T, Cheng CY (2014). Global prevalence of glaucoma and projections of glaucoma burden through 2040: A systematic review and meta-analysis. Ophthalmology.

[2] Kyari F, Abdull MM, Bastawrous A, Gilbert CE, Faal H (2013). Epidemiology of glaucoma in Sub-Saharan Africa: Prevalence, incidence and risk factors. Middle East Afr J Ophthalmol.

[3] Naidoo K, Gichuhi S, Basáñez MG, Flaxman SR, Jonas JB, Keeffe J (2014). Prevalence and causes of vision loss in sub-Saharan Africa: 1990-2010. Br J Ophthalmol.

[4] Kyari F, Abdull MM, Bastawrous A, Gilbert CE, Faal H (2013). Epidemiology of glaucoma in Sub-Saharan Africa: Prevalence, incidence and risk factors. Middle East Afr J Ophthalmol.

[5] Abdull MM, Gilbert CC, Evans J (2015). Primary open angle glaucoma in northern Nigeria: Stage at presentation and acceptance of treatment. BMC Ophthalmol.

[6] Josephine AB, Feyisayo AB, Grace BB, Medinat BM, Ibidapo OO (2013). Patient refusal of glaucoma surgery and associated factors in Lagos, Nigeria. Middle East Afr J Ophthalmol.

[7] Smith AF, Negretti G, Mascaro A, Bokre D, Baker H, Dhalla K (2018). Glaucoma Control Strategies in Sub-Saharan Africa: A Review of the Clinical and Health Economic Evidence. Ophthalmic Epidemiol.

[8] Realini T, Olawoye O, Kizor-Akaraiwe N, Manji S, Sit A (2018). The rationale for selective laser trabeculoplasty in Africa. Asia-Pacific J Ophthalmol.

[9] Gazzard Gus, Konstantakopoulou Evgenia, Garway-Heath David, Garg Anurag, Vickerstaff Victoria, Hunter Rachael, Ambler Gareth, Bunce Catey, Wormald Richard, Nathwani Neil, Barton Keith (2019). Selective laser trabeculoplasty versus eye drops for first-line treatment of ocular hypertension and glaucoma (LiGHT): a multicentre randomised controlled trial. Lancet.

[10] Bowman RJC, Kirupananthan S (2006). How to manage a patient with glaucoma in Africa. Community Eye Heal J.

[11] Yorston D, Khaw PT (2001). A randomised trial of the effect of intraoperative 5-FU on the outcome of trabeculectomy in east Africa. Br J Ophthalmol.

[12] Kyari F, Adekoya B, Abdull MM, Mohammed AS, Garba F (2018). The current status of glaucoma and glaucoma care in sub-Saharan Africa. Asia-Pacific J Ophthalmol.

[13] Resnikoff S, Lansingh VC, Washburn L, Felch W, Gauthier TM, Taylor HR (2020). Estimated number of ophthalmologists worldwide (International Council of Ophthalmology update): Will we meet the needs?. Br J Ophthalmol.

[14] Egbert PR (2002). Glaucoma in West Africa: A neglected problem. Br J Ophthalmol.

[15] Dean W, Gichuhi S, Buchan J, Matende I, Graham R, Kim M (2019). Survey of ophthalmologists-in-training in Eastern, Central and Southern Africa : A regional focus on ophthalmic surgical education [ version 1 ; peer review : awaiting peer review ]. Wellcome Open Res.

[16] Annoh R, Banks LM, Gichuhi S, Buchan J, Makupa W, Otiti J (2021). Experiences and Perceptions of Ophthalmic Simulation-Based Surgical Education in Sub-Saharan Africa. J Surg Educ.

[17] Daly MK, Gonzalez E, Siracuse-Lee D, Legutko PA (2013). Efficacy of surgical simulator training versus traditional wet-lab training on operating room performance of ophthalmology residents during the capsulorhexis in cataract surgery. J Cataract Refract Surg.

[18] Balasopoulou A, Kokkinos P, Pagoulatos D, Plotas P, Makri OE, Georgakopoulos CD (2017). Symposium Recent advances and challenges in the management of retinoblastoma Globe - saving Treatments. BMC Ophthalmol.

[19] Belyea DA, Brown SE, Rajjoub LZ (2011). Influence of surgery simulator training on ophthalmology resident phacoemulsification performance. J Cataract Refract Surg.

[20] Feudner EM, Engel C, Neuhann IM, Petermeier K, Bartz-Schmidt KU, S P (2009). Virtual reality training improves wet-lab performance of capsulorhexis: results of a randomized, controlled study. Graefes Arch Clin Exp Ophthalmol.

[21] Dean WH, Buchan J, Gichuhi S, Philippin H, Arunga S, Mukome A (2021). Simulation-based surgical education for glau-coma versus conventional training alone: the GLAucoma Simulated Surgery (GLASS) trial. A multi-centre, multicountry, randomised controlled, investigator-masked educational intervention efficacy trial in Kenya, South Africa, Tanzania, Uganda and Zimbabwe. Br J Ophthalmol.

[22] Dean W, Burton M, Arunga S, Buchan J, Cook C, Gichuhi S (2017). The Simulated Ocular Surgey (SOS) Trials: Randomised-Controlled Trials Comparing Intense Simulation-Based Surgical Education For Cataract and Glaucoma Surgery To Conventional Training Alone in East and Southern Africa.

[23] Peyton J (1998). Teaching and learning in medical practice.

[24] Porteous AM, Ahmed F (2018). A novel wet-lab teaching model for trabeculectomy surgery. Eye.

[25] Dean WH, Buchan J, Admassu F, Kim MJ, Golnik KC, McNaught A (2019). Ophthalmic simulated surgical competency assessment rubric (Sim-OSSCAR) for trabeculectomy. BMJ Open Ophthalmol.

[26] Jonas JB, Rabethge S, Bender HJ (2003). Computer-assisted training system for pars plana vitrectomy. Acta Ophthalmol Scand.

[27] Solverson DJ, Mazzoli RA, Raymond WR, Nelson ML, Hansen EA, Torres MF (2009). Virtual reality simulation in acquiring and differentiating basic ophthalmic microsurgical skills. Simul Healthc.

[28] Benjamin L (2014). 25th RCOphth congress, president’s session paper: 25 years of progress in surgical training. Eye.

[29] Staropoli PC, Gregori NZ, Junk AK, Galor A, Goldhardt R, Goldhagen BE (2018). Surgical Simulation Training Reduces Intraoperative Cataract Surgery Complications among Residents. Simul Healthc.

[30] Alaker M, Wynn G, Arulampalam T (2016). Virtual reality training in laparoscopic surgery: A systematic review & meta-analysis. Int J Surg.

